# No Genomic Signatures Were Found in *Escherichia coli* Isolates from Camels With or Without Clinical Endometritis

**DOI:** 10.3390/vetsci12050457

**Published:** 2025-05-10

**Authors:** Haitham Elbir

**Affiliations:** Camel Research Center, King Faisal University, 400 Al-Hasa, Hofuf 31982, Saudi Arabia; helbir@kfu.edu.sa

**Keywords:** she-camel, endometritis infection, evolution virulence, *Escherichia coli* genome

## Abstract

Clinical endometritis is a leading cause of infertility in she-camels. We commonly isolate *E. coli* from camel uteri with and without endometritis during our routine diagnosis of conception failure. From an epidemiological standpoint, it is critical to know if certain *E. coli* genotypes and virulence factors are specifically associated with endometritis. Thus, we aimed to compare the abundance of virulence elements and genotypes in uterine *E. coli* from camels with and without endometritis and understand their evolution. For this investigation, we retrieved data from the genomes of 28 *E. coli* isolates from humans, cats, dogs, horses, cows, and birds and 14 sequenced genomes of camel uterine *E. coli* isolates. We found no specific *E. coli* genotype or virulence factor associated with endometritis. Instead, multiple genotypes and high genomic diversity were observed. Moreover, horizontal gene transfer driven by genomic islands and plasmids contributed to the genetic diversity of the isolates, resulting in the acquisition of virulence genes, metabolic characteristics, and antibiotic resistance determinants to trimethoprim, sulfonamide, streptomycin, and tetracycline. Additionally, the phylogenetic position of the *E. coli* isolates from camel uteri suggests that they originated from intestinal strains. In conclusion, there was no evidence of *E. coli* specialization, and *E. coli* alone may not be able to develop endometritis, as other factors are required. Also, we elucidated the mechanism behind the diversity of the gene repertoire of *E. coli* isolated from camel uteri. These findings provide insight into the evolutionary origins of *E. coli* isolates from camel uteri.

## Simple Summary

*Escherichia coli* is frequently isolated from camels with endometritis. In this study, we compare the virulome, genotypes, resistome, and mobilome of uterine *E. coli* collected from camels with and without clinical endometritis for the first time. Through genomic analysis, we found no specific *E. coli* genotype or virulence factor associated with endometritis. Moreover, we elucidated the contribution of horizontal gene transfer to the diversity of the gene repertoire of *E. coli*.

## 1. Introduction

Clinical endometritis, which is an inflammation of the endometrium in response to colonization and growth of microorganisms [1], is a leading cause of infertility in she-camels, causing animal distress and economic losses to the camel industry. In Saudi Arabia, clinical endometritis accounts for 43.8% of the conditions influencing the reproductive performance of camel herds [2]. *Escherichia coli* was classified based on the site of infection into intestinal *E. coli* and extraintestinal *E. coli* [3]. The intestinal pathogenic *E. coli* was further divided into numerous pathotypes according to the existence of certain virulence genes and pathology, such as enterohemorrhagic *E. coli* (EHEC). [4], enteropathogenic *E. coli* (EPEC) [5], and enterotoxigenic *E. coli* (ETEC) strains [6]. Also, the extraintestinal pathogenic *E. coli* was further classified into several pathotypes, such as uropathogenic *E. coli* (UPEC) [7], avian pathogenic *E. coli* (APEC) [8], and endometrial pathogenic *E. coli* (EnPEC) [9].

Despite that several different bacterial species were isolated from clinical endometritis, *E. coli* was frequently isolated from endometritis [10,11,12,13], and an in vivo study showed that *E. coli* and *Trueperella pyogenes* can develop endometritis in cows [14]. These findings led to the notion that *E. coli* is probably involved in developing endometritis. The potential role of uterine *E. coli* was further investigated through profiling their virulence factors. In a PCR-based investigation, Bicalho et al. [15] found six virulence factors (*fimH*, *astA*, *cdt*, *kpsMIII*, *ibeA*, and *hlyA*) associated with metritis in cows. Another PCR study by Kassé et al. [16] found two virulence factors (*hra1* and *kpsMTII*) associated with metritis in cows. In contrast, a recent genome-based investigation could not identify any virulence factors associated with metritis [17]. Given the contradictory results indicated above, it is currently too early to assess the pathogenic potential of uterine *E. coli* by only profiling its virulence factors. In addition, since some *E. coli* strains cause diseases in humans but not in animals that act as reservoirs [18], it is not necessary to expect the same results that were observed in *E. coli* from cows with metritis to be observed in *E. coli* isolated from camels with endometritis.

Mobile genetic elements (MGEs), including genomic islands and plasmids, play a major role in horizontal transfer of genes that contribute to the diversity of species [19,20]. Large-scale comparison of *E. coli* isolates is challenging due to the high genetic diversity caused by gene gain carried by mobile genetic elements. Such a degree of diversity can make it difficult to ascertain to what extent virulence factors or other genetic signatures are associated with *E. coli* from camels with or without endometritis. Additionally, the impact of HGT on uterine *E. coli* genetic diversity from cows or camels remains yet to be investigated. Given that we frequently isolate *E. coli* from the uteri of camels with and without endometritis. The main objectives of the present study are (1) to determine whether uterine *E. coli* virulence genes and genotypes are associated with camel endometritis, (2) to clarify the origin and phylogenomic relationship of camel uterine *E. coli* isolates, and (3) to elucidate the impact of mobile genetic elements on gene repertoire diversity of camel uterine *E. coli* isolates.

## 2. Materials and Methods

### 2.1. Sample Collection

The current study received permission from the Research Ethics Committee of King Faisal University (HAPO-05-HS-003). The study was performed in the Camel Research Center, King Faisal University, in Al-Hasa province, Saudi Arabia. A total of 14 *E. coli* isolates were chosen from a group of bacteria that had previously been recovered from a she-camel’s uterus during routine diagnosis. Clinical endometritis was described by Ali and Derar [1] as the appearance of vaginal discharges following postpartum due to inflammation of the endometrium. Two groups of camels were defined based on Ali and Derar’s [1] criteria as camels without endometritis (7 *E. coli* isolates) and camels with clinical endometritis (7 *E. coli* isolates). The age range of the she-camels in our study was 6–7 years old, and they had a history of unsuccessful conception despite repeatedly mating with fertile camels. They only gave birth once, and there were no recorded abortions. The camels were all in good health and received no treatment.

Transrectal palpation and transrectal ultrasound of the uterus utilizing a 7.5 MHz linear transducer probe (Aloka, Co., Ltd., Tokyo, Japan) were part of the reproductive tract inspection for confirmation of the presence of fluids and pus in the uterus. Additionally, the cervix and vagina were inspected. For sample collection from the uterus, acriflavine 0.1% was used to disinfect the perineum of the camels. Then, a double-guarded sterile swab (Kruuse, Langeskov, Denmark) was inserted through the vagina and cervix into the uterine lumen. Inside the uterus, the cotton swab was taken out of the double-guard tube, rubbed on the endometrium of the uterus, and then returned back into the guard before removing it from the uterine lumen. Sterile surgical gloves were used in sample processing. The swabs were immediately transferred within one hour to the laboratory. Swabs were opened and rolled onto 5% sheep blood agar and were incubated aerobically at 37 °C for 48 h.

### 2.2. Genome Sequencing

The *E. coli* DNA was isolated from culture using the Wizard genomic DNA kit (Promega Biotech AB, Madison, WI, USA) following the company-provided procedure. DNA libraries were constructed using the TruSeq Nano DNA Library Preparation Kits (Illumina, San Diego, CA, USA) and sequenced on Illumina’s NovaSeq platform by Macrogen Inc. (Seoul, Republic of Korea). Fastqc 0.11.8 [21] was used to check the read sequence quality. Then, the read sequence was trimmed with fastp 0.20.0 [22]. Next, sequence reads were assembled by SPAdes 3.15.4 [23], and PlasmidSPAdes mode using the -plasmid option was used to only assemble plasmids from WGS. For downstream analysis, only scaffolds longer than 500 base pairs and with a coverage value greater than 10% were kept. Each *E. coli* assembly was annotated using Prokka 1.14.6. The PTS, ABC transporters, and metabolic KEGG modules for every genome were reconstructed using the Kyoto Encyclopedia of Genes and Genomes (KEGG) website [24].

#### 2.2.1. Identification and Typing of Isolates

Swabs were rolled onto 5% sheep blood agar followed by incubation for 48 h at 37 °C.

Then, they were initially classified as *E. coli* by 16S rRNA sequencing and lactose fermentation testing on the MacConkey agar.

The *E. coli* str. K-12 substr. MG1655 genome sequence was designated as the reference genome by GenBank. Therefore, for confirmation of identification, genomic similarity against the reference sequence of *E. coli* str. K-12 substr. MG1655 was estimated using the standalone version of OrthoANI (OAT v. 1.40 using blastn 2.13.0+) [25]. The isolates were identified as *E. coli* when their OrthoANI value was within 95–96% [26]. We determined multilocus sequence types (MLSTs) of *E. coli* genome sequences by using the online tool PubMLST following Achtman criteria [27]. The EzClermont command-line tool was used to phylotype each genome [28]. For serotype, we utilized the online tool SerotypeFinder [29].

#### 2.2.2. Mobilome and Virulome

Prophages were screened in the genomes utilizing PHASTER [30]. Genomic islands (GIs) are often made up of foreign DNA pieces acquired through horizontal transfer. The online tool IslandViewer 4 was used to detect GIs with default settings [31]. In general, the ORFs identified in GI areas were regarded as horizontally transmitted genes. The online tool IslandCompare (v1.0) was used for comparisons of genomic islands across the genomes of *E. coli* isolates [32].

For the detection of virulence factors, insertion sequences, and plasmid replicons, the online tool MobileElementFinder was used [33]. The online ICEfinder [34] was used for prediction of Integrative Conjugative Elements [ICEs], which carry an integrase gene, a relaxase gene, and T4SS gene clusters.

#### 2.2.3. Phylogenomic

In the first approach, only single-copy core genes shared by the outgroup *Escherichia albertii* (CP117568.1) and the 14 *E. coli* isolates were used for phylogenomic tree inference. GET_HOMOLOGUES scripts [35] were used to determine core genes with a query coverage of >80% and a sequence similarity of >85%. Based on protein sequence analysis, 2767 core genes were identified. To more effectively differentiate distinct *E. coli* isolates, the nucleotide sequence was employed rather than the protein sequence. Each core gene sequence was aligned individually using MAFFT v7.490 with default settings [36], and IQ-TREE v2.2.0 [37] was utilized for tree reconstruction after the obtained alignments were concatenated. Tree branch support was determined by 1000 replicates, and IQ-TREE ModelFinder was used to predict the best model. iTOL v7 [38] was used to visualize the resultant tree.

In the second approach, pangenome content was analyzed by GET_HOMOLOGUES scripts and yielded a binary matrix derived from the presence/absence of genes. The resulting binary matrix was used for tree reconstruction with IQ-TREE v2.2.0. iTOL v7 was used to visualize the resultant tree.

Finally, to understand the evolution of camel uterine isolates, we analyzed the phylogenetic position of 14 camel uterine *E. coli* isolates with 28 *E. coli* strains from humans, horses, dogs, cats, birds, and cows. The 28 *E. coli* strains were selected from the genomes available in the GenBank database and included the following strain names and GenBank accessions: APECO1 (CP000468.1), APECO18 (CP006830.1), BW2952 (CP001396.1), CB9615 (CP001846.1), CD306 (CP013831.1), DH1 (CP001637.1), ECOL (AASAOI010000003.1), JJ1886 (CP006784.1), KCJ3816 (NZ_PCGF01000082.1), KCJ3820 (NZ_PCGI01000188.1), KCJ3821 (NZ_PCGJ01000171.1), KCJ3822 (NZ_PCGK01000077.1), KCJ3823 (NZ_PCGL01000169.1), KCJ3824 (NZ_PCGM01000258.1), KCJ3857 (NZ_PCGO01000215.1), KCJ3858 (NZ_PCGP01000138.1), KCJ3859 (NZ_PCGQ01000254.1), KCJ4030 (NZ_SMUL01000002.1), KCJ4031 (NZ_SMUM01000008.1), KCJ4032 (NZ_SMUN01000007.1), KCJ4033 (NZ_PGUE01000050.1), KCJ4034 (NZ_PGUF01000236.1), KG-2 (NZ_PVOH01000052.1), KG-8 (NZ_MPAY01000069.1), MDS42 (AP012306.1), PairA_ch_2 (NZ_PXVX01000017.1), RM12579 (CP003109.1), TW14359 (CP001368.1). Only single-copy core genes shared by 42 *E. coli* genomes (14 from camels, 7 from humans, 1 from dogs, 1 from cats, 2 from birds, 1 from horses, and 16 from cows) were used for phylogenomic tree inference. Each core gene sequence was aligned individually using MAFFT v7.490 with default settings [36], and IQ-TREE v2.2.0 [37] was utilized for tree reconstruction after the obtained alignments were concatenated. Tree branch support was determined by 1000 replicates, and IQ-TREE ModelFinder was used to predict the best model. iTOL v7 was used to visualize the resultant tree.

#### 2.2.4. Antibiotic Susceptibility Testing

The Kirby–Bauer disk diffusion method was used to determine antibiotic susceptibility in accordance with the European Committee on Antimicrobial Susceptibility Testing, Clinical breakpoints [39]. Each isolate was tested for its susceptibility to gentamicin (10 µg per disk), ceftazidime/avibactam (50 µg), amikacin (30 µg), ceftaroline (30 µg), cefazolin (30 µg), ampicillin/sulbactam (20 µg), amoxicillin/clavulanic (30 µg), streptomycin (10 µg), ceftazidime (30 µg), ceftiofur (30 µg), meropenem (10 µg), cefotaxime (30 µg), ceftriaxone (30 µg), and tetracycline (15 µg). The command-line AMRFinderPlus 4.0.3 software [40] was employed to detect the existence of point mutations and resistance determinants. Multi-drug resistance (MDR) can be described as non-susceptibility to at least one antibiotic in three or more classes [41].

#### 2.2.5. Statistical Analysis

The prevalence of antimicrobial resistance genes and virulence genes was reported as a percentage. Statistical analysis for prevalence of phylogroups, genotypes, virulence factors, and antibiotic resistance was performed using Fisher’s exact two-tailed test. *p*-values ≤ 0.05 were used as a significance threshold.

## 3. Results

### 3.1. ANI, Phylogroups, Serotype, and MLST Analysis

To determine how closely related the isolates are, we calculated pairwise ANI between every uterine isolate versus reference *E. coli* str. K-12 strain. The ANI values across clinical endometritis (CE) isolates and non-CE isolates varied from 98.5 to 99.6% and 98.5 to 98.6%, respectively (Table 1). Among the non-CE isolates, the pairwise ANI value revealed that 90.5% of the isolates had an ANI value > 99%, whereas only 9.5% had an ANI value > 98 and <99%. The pairwise ANI value across CE isolates revealed that only 71.4% of the isolates had ANI values > 98 and <99%, whereas 28.5% of the isolates had ANI values > 99% (Table S1). The CE isolates had 3220 and 5734 genes in their core and pangenome, respectively, whereas non-CE isolates have 3520 and 6052 genes in their core and pangenome, respectively.

Phylogroup analysis clustered the 14 uterine *E. coli* isolates into three phylogroups (Table 1). The most frequent phylogroup was phylogroup B1 (79%), followed by phylogroup A (14%) and phylogroup C (7%). The phylogrouping assigned all non-CE isolates to phylogroup B1, while CE isolates were assigned to phylogroups B1, A, and C. The results showed that phylogroup B1 is dominant in both groups.

The MLST typing showed different sequence types for the uterine isolates (Table 1). Of the fourteen isolates, eleven *E. coli* isolates can be grouped into nine different STs, while the remaining isolates, 2649, 1922big, and 2613, showed the closest profile to ST13358, ST707, and ST2520. The only most frequent ST was ST58. We found two CE isolates and one non-CE isolate assigned to ST58. CE isolates were grouped into six STs, while non-CE isolates were grouped into five STs. The STs reported here, comprising ST58, ST88, ST173, ST295, ST2164, and ST17, are pathogenic clonal lineages reported globally according to literature.

The serotyping was also performed and clustered the isolates into 14 different serotypes. O antigen was not detectable for isolates 2649 and 4436A. Isolates assigned to the ST58 genotype were found to have different serotypes, including O50:H8, O9:H12, and O8:H21. The serotype reported here, comprising O113:H21, a foodborne pathogen that is Shiga toxin-producing *E. coli.* Therefore, no association between the isolate phylogroups, serotype, MLST, and clinical status of camels was identified.

### 3.2. Phylogenomic Analyses

**Figure 1:**
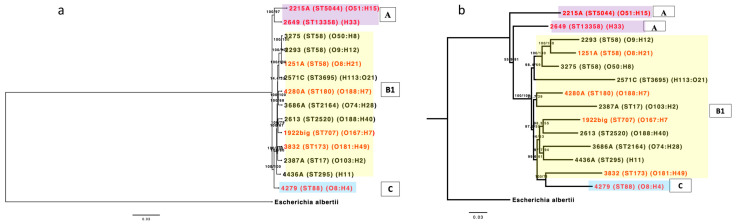
(**a**) Phylogenomic tree inferred from core genes. The colored boxes represent phylotypes A, B1 and C. The numbers in the branch are SH-aLRT support and ultrafast bootstrap support. Each isolate’s ST type and serotype are mentioned next to the isolate name. (**b**) Hierarchical grouping of the seven CE (red) and seven non-CE *E. coli* genomes based on gene presence/absence. The colored boxes represent phylotypes A, B1 and C. The numbers in the branch are SH-aLRT support and ultrafast bootstrap support. Each isolate’s ST type and serotype are mentioned next to the isolate name.

Core or whole-genome sequence-based phylogeny is required for higher resolution among strains of the same species. Thus, from the 2767 core genes, we inferred a phylogenomic tree that clustered the 14 isolates into three different clades. Two CE isolates from phylogroup A formed one clade, and another CE isolate from phylogroup C formed one clade. The remaining isolates from phylogroup B1, which includes both CE and non-CE isolates, were grouped into a single clade. Furthermore, CE isolates and non-CE isolates with the ST58 genotype grouped together (Figure 1a). In addition to the aforesaid phylogenomic tree, we also constructed a phylogenomic tree from the presence and absence matrix of the 10,410 pan-genes. Again, three clades were formed by the isolates. Two distinct clades were formed from two CE isolates. Another clade contained the remaining CE and non-CE isolates (Figure 1b). The topology of pangenome-derived tree and core gene tree are different regarding the clustering of 4279, 3832, 4436A, 2387A, and 4280A isolates. Moreover, the structure of the clade and the phylogroup differs; for example, the pangenome-generated tree no longer distinguishes the isolates of phylotype B1 from *E. coli* 4279 in phylotype C. This suggests that some isolates are more alike in terms of the sequence of their core genes than in terms of their accessory genes. Since there is no evolutionary difference between CE isolates and non-CE isolates, the isolate clustering described above did not correlate with clinical state.

**Figure 2:**
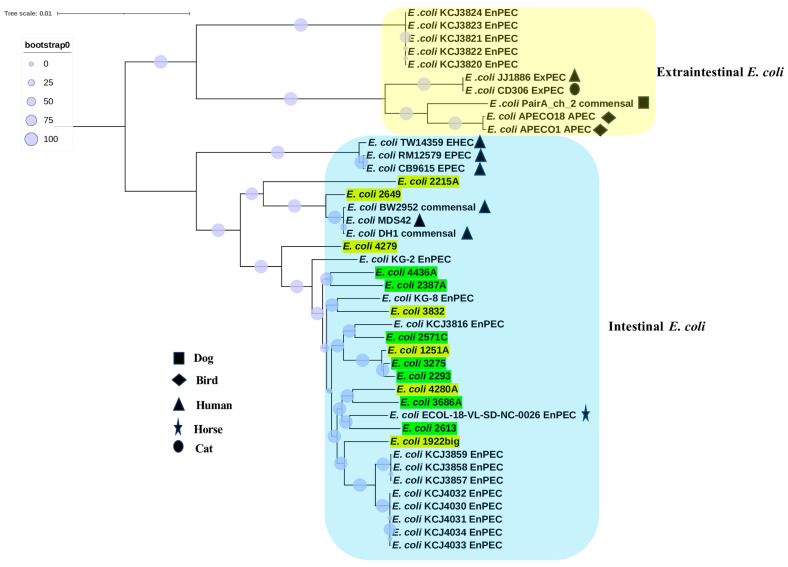
Phylogenomic tree inferred from core genes. The yellow color indicates CE isolates, while the green color indicates non-CE isolates. EnPEC is *E. coli* from cow uteri.

Finally, to understand the evolution of camel uterine isolates, we analyzed the phylogenetic position of 14 camel uterine *E. coli* with 28 *E. coli* strains from human, horse, dog, cat, bird, and cow sources (Figure 2). The phylogenomic tree was constructed based on 2153 core genes and revealed two distinct clades that represented intestinal and extraintestinal strains of *E. coli*. The camel *E. coli* isolates grouped with intestinal *E. coli*.

### 3.3. Antibacterial Susceptibility Profile

The CE isolates and non-CE isolates differed in their phenotypic antibacterial susceptibility as determined by the disk diffusion method. The most prevalent resistance found in CE isolates was by far tetracycline (57.1%), followed by streptomycin (28.5%), sulfonamide (28.5%), ceftazidime (14.3%), and trimethoprim (14.3%). The rest of the antibiotics (gentamicin, ceftazidime/avibactam, amikacin, ceftaroline, cefazolin, ampicillin-sulbactam, amoxicillin-clavulanic, ceftiofur, meropenem, cefotaxime, and ceftriaxone) were effective against these *E. coli* isolates. As for the non-CE isolates, the most prevalent resistance was by far ceftazidime (28.5%), followed by tetracycline (14.3%), streptomycin (14.3%), and sulfonamide (14.3%). The rest of the antibiotics were effective. The number of isolates that exhibited an MDR pattern (resistance to ceftazidime, tetracycline, and streptomycin) was one in CE isolates and non-CE isolates (Table 2).

After that, we analyzed the resistomes of CE and non-CE isolates (Table 2). The findings showed that 57.1% of CE isolates and one non-CE isolate had the tetracycline resistance determinant *tetA*. Other resistance determinants included streptomycin resistance determinants *strA* and *strB*, which were found in 28.6% of CE isolates and in one non-CE isolate. The trimethoprim resistance determinant *dfrA1* was detected in one CE isolate, whereas *dfrA5* was detected in one non-CE isolate. The sulfonamide resistance determinant *sul2* was detected in 28.6% of CE isolates and in one non-CE isolate table. For CE isolate 4280A and non-CE isolates 2293 and 2613, we were unable to determine the genetic basis underlying the ceftazidime resistance phenotype. The prevalence of resistance determinants among CE isolates, which was 57%, was not significantly higher than that of non-CE isolates, which was 14.3% (*p* > 0.05).

### 3.4. Virulome

To explore differences in pathogenicity potential, all CE isolates and non-CE isolates were screened for virulence factors, finding 58 different virulence genes categorized into groups including adhesin, toxin, iron uptake, protectin, invasion, and others (Table 3). The repertoire of virulent genes varied from 12 to 33 in CE isolates and 13 to 26 in non-CE. The common virulence factors identified in all CE isolates and non-CE isolates included adhesin (*yehA, yehB, yehC, nlpI,* and *csgA*), toxin (*hlyE*), and terC. Despite there being no virulence factors present in all non-CE isolates, protectins (*hha, espA, espB, espF, espJ*, and *etpD*), toxin (stx2b-O174-031), iron uptake (*ireA*), and invasion (*tia*) virulence factors were often present (<43%) in non-CE isolates and absent in all CE isolates. In contrast, the following virulence factors: toxin (*hlyF* and *senB*), invasion (*AslA*), iron uptake (*fyuA, iroN, irp2, iucC, iutA*, and *sitA*), and protectin (*capU*, *cia*, *cma*, *colE7*, *cvaC*, *mchBCF*, and *mcmA*) were frequently present (<43%) in CE isolates and absent from non-CE. There was no significant correlation (*p* > 0.05) between the occurrence of virulence factors and whether or not clinical endometritis was present.

For further investigation of pathogenicity, we screened the genome for toxin–antitoxin systems, which have become recognized as potential virulence factors (Table S2). The TA systems found in our isolates were related to biofilm formation, persistence, abortive infection, and acidic pH response. The number of TA loci per uterine *E. coli* strain varied from 13 to 17 in CE isolates, while the number varied from 15 to 18 in non-CE isolates. The core TA loci for the 14 uterine *E. coli* consist of seven TA loci. As for the non-core TA systems, the difference in prevalence was not significant (*p* > 0.05) between CE isolates and non-CE isolates.

### 3.5. Comparative Analysis of Functional Characteristics

To explore the differences in functional features between *E. coli* genomes from the uterus with and without clinical endometritis, we reconstructed the PTS and ABC transporters and metabolic KEGG modules. A detailed comparison of the features for CE isolates and non-CE isolates revealed that they share several types of PTS and ABC transporters. In contrast, there is no PTS or ABC transporter that is present in all CE isolates and missing from all non-CE isolates. Instead, there are four PTS that differ in prevalence between CE isolates and non-CE isolates. Of these, the PTS system involved in D-glucosaminate transport was detected in 29% of the CE isolates and detected in only 14.3% of the non-CE isolates. Galactitol was detected in 29% of the CE isolates and in only 43% of non-CE isolates. The sorbose transporter is present in 14.3% of CE isolates, while the sucrose transporter is present in 14.3% of non-CE isolates.

As for the ABC transporter, the ABC iron transporter encoded by *FecBCDE* and *AfuABC* was frequently present in the CE isolates (57.1%, 29%) and found in only 29% and14.3% of non-CE isolates. The iron (II) manganese transporter encoded by the genes *SitABCD* was frequently present in the CE isolates (29%) and was absent in non-CE isolates.

As for the metabolic KEGG modules, all isolates share 90 metabolic KEGG modules. The number of shared metabolic KEGG modules in the genome of CE isolates and non-CE isolates was 90 and 97, respectively. The number of modules per isolate ranged from 93 to 100 and 97 to 100 modules in the CE isolates and non-CE isolates, respectively. As for the differences in the metabolic potentials, detailed analysis revealed 13 modules that differ in prevalence between CE isolates and non-CE isolates. Seven out of the 13 modules were 100% prevalent in non-CE isolates, while their prevalence was between 100% and 29% in CE isolates. The above-mentioned seven modules included M00697 for multidrug resistance efflux pump MdtEF-TolC, M00878 for phenylacetate degradation, M00027 for GABA (gamma-aminobutyrate) shunt, M00761 for undecaprenylphosphate alpha-L-Ara4N biosynthesis, M00958 for adenine ribonucleotide degradation, M00959 for guanine ribonucleotide degradation, and M00982 for methylcitrate cycle.

Of all the isolates, the CE isolate 2215A had the fewest modules (93 KEGG modules) as it lacks the following modules: M00697 for multidrug resistance efflux pump MdtEF-TolC, the module M00958 for adenine ribonucleotide degradation, and the module M00959 for guanine ribonucleotide degradation. These findings might suggest that CE isolate 2215A is unable to utilize adenine and guanine as nitrogen sources for growth. Furthermore, CE isolate 2251A lacks the methylcitrate cycle module M00982, indicating that it is unable to use propionate as its only carbon source. Additionally, it lacks the M00555 module for the production of betaine, which is triggered by osmotic stress. Finally, it lacks the KEGG module for degradation of homoprotocatechuate and phenylacetate, which are derived from a variety of substrates in the environment. Degradation of these compounds enables bacteria to use this compound as a carbon and energy source for growth.

### 3.6. Mobilome Analysis

The mobilome is the complete set of genes that could have been obtained by horizontal gene transfer (HGT). We identified an array of mobile genetic elements (MGEs), such as plasmids, genomic islands, insertion sequences, and prophages (Table S3). The plasmids of CE isolates and non-CE isolates were compared by prevalence rate, replicon type, sequence similarity, and resistance determinant carriage. The prevalence of plasmids was 71.4% in CE isolates and 100% in non-CE isolates. All uterine isolates, with the exception of those that contained no plasmids, carried one to eight plasmids. The plasmids from CE isolates are distant from non-CE isolates, but some plasmids from CE isolates share (<40% of nucleotide identity) with plasmids from non-CE isolates. Eleven and nine replicon types were found in CE isolates and non-CE isolates, respectively. The following six replicon types: Col(MG828), IncFIA, IncFIB(AP001918), IncFII, IncFII(pCoo), and IncFII(pHN7A8) were detected in at least one isolate from both groups. In contrast, the replicon types ColpVC, IncX1and IncY were not detected in CE isolates, while the replicon types IncI1, IncQ1, IncR, IncFII(29), and Col156 were not detected in non-CE isolates. Isolates belonging to the same ST had their plasmids compared. None of the three ST58 isolates carried similar plasmid sequences, but two of the three isolates had the same plasmid type, IncFIB(AP001918). We next compared the antibiotic-resistant determinants among both groups. For the CE isolate 3832, the antibiotic-resistant determinants *dfrA5* and *tetA* are carried by ICE integrated in plasmid type IncFII. For the CE isolate 4279, the *sul2-strA-strB* and *tetA* locus, are carried by ICE integrated in plasmid type IncFII. The *tetA* antibiotic-resistant determinant of CE isolate 1922big is carried by ICE integrated into plasmid type IncI1. The *sul2-strA-strB-dfrA1-tetA* locus of CE isolate 4082A is carried by ICE integrated in the chromosome. As for the non-CE isolate 2613, the antibiotic-resistant determinants *sul2-strA-strB-dfrA1-tetA* locus are integrated in the chromosome and were not associated with ICE. Notably, the CE isolate 4279 and the CE isolate 3832 plasmids are similar (95% coverage and 99% identity). The plasmid of CE isolates 4279 and 3832 had already been found globally in strains of several *E. coli*. The closest GenBank entry was *E. coli* plasmid pME13 (accession MT868888.1), having homology of 98% coverage and 99% identity.

**Figure 3:**
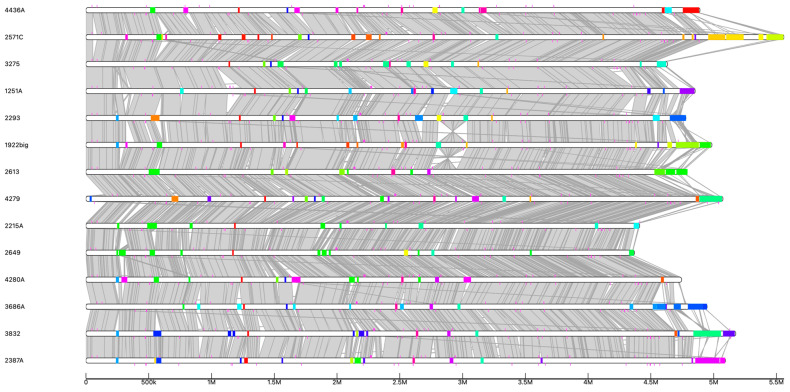
White bars represent genomes, whereas colored boxes represent genomic islands. Gray indicates genome alignments.

Apart from plasmids, GIs are mobile elements that have been demonstrated to improve bacterial adaptation into different habitats and may encode genes linked to pathogenicity and antibiotic resistance. Uterine *E. coli* isolates exhibited varying numbers of pathogenic genomic islands ranging from one to five and one to six in *E. coli* from CE and non-CE, respectively (Figure 3). Notably, we found a percentage of 10.1%, 9.1%, 11.1%, 14.1%, 14.8%, 15.7%, and 18.0% of genes were associated with the pool of GIs in CE isolates 2649, 2215A, 4280A, 1251A, 1922big, 4279, and 3832, respectively (Table S4). In the case of non-CE isolates 3275, 2293, 2613, 4436A, 3686A, 2387A, and 2571C, we found a percentage of 11.9%, 15.0%, 13.6%, 15.1%, 15.4%, 16.85 and 20.4% of genes were associated with the pool of GIs, respectively. After a detailed inspection of GIs, the percentage of virulence genes carried by GIs varied between 31 and 65% in non-CE isolates and between 33.3 and 78.8% in CE isolates. GIs harbored all the antibiotic-resistant genes found here. Additionally, GIs carried six of the twenty-one loci for TAs. The KEGG modules found in GIs included M00918 aerobactin biosynthesis in CE isolates 3832 and 4279. M00793 dTDP-L-rhamnose biosynthesis in non-CE isolate 3275 and in CE isolates 2215A and 2649. Along with KEGG modules, the GIs in non-CE isolates 2293 and 4436A as well as in CE isolates 3832, 4280A, 1251A, and 4279 harbored the iron ABC transporter encoded by *FecBCDE* genes. Also, the GIs in CE isolate 1251A and non-CE isolate 2293 harbored the iron ABC transporter encoded by *AfuABC*. Lastly, the GIs in CE isolate 1251A and non-CE isolates 4436A and 2571C had the PTS transporter for ascorbate, whereas the GIs in non-CE isolate 4436A carried the PTS transporter for sucrose. Regarding the other MGEs, CE isolates exhibited a range of prophages from one to seven, whereas non-CE isolates showed a range of two to eight (Table S3). A number of insertion sequences that are specific to CE isolates or non-CE isolates were also detected. However, their frequency was not statistically significant. In summary, no mobile genetic elements linked to clinical state were found.

## 4. Discussion

This study describes, for the first time, the phylogeny, genotypes, virulome, resistome, and mobilome of *E. coli* isolated from the uterus of camels with and without clinical endometritis.

Firstly, our results showed that *E. coli* does not specialize in causing endometritis, given that there are no particular genotypes or virulence genes linked to endometritis in camels. Our findings revealed high genetic diversity across the CE and non-CE isolates, with the ST58 genotype being shared by both groups, which further shows the non-specialization of *E. coli* associated with endometritis. Furthermore, the high genetic diversity observed in the isolates resulted in a phylogeny that did not exhibit any clustering based on the clinical status of camels. Instead, the majority of CE and non-CE isolates clustered together in the phylogenomic tree derived from the core and pangenome rather than splitting the isolates into two distinct clades. This result contradicts a prior PCR-based study in cows that found a connection between some *E. coli* virulence factors and uterine infection [15], and it is consistent with a recent genome-based investigation in cows with metritis [17].

Secondly, we wanted to compare the intra-species genomic similarity, phylogroup, and evolution between the populations of CE isolates and non-CE isolates, which revealed differences. For intra-species genomic similarity, whole-genome similarity metrics such as ANI help us to compute pairwise ANI values among isolates. According to ANI values, non-CE isolates are genomically more similar to one another than CE isolates. Additionally, the core genome of non-CE isolates is larger than that of CE isolates. Another difference in terms of phylogroup was that CE isolates belonged to phylogroups B1, A, and C, while non-CE isolates belonged to phylogroup B1. This result is consistent with previous findings in cows and horses, where the majority of uterine isolates were from phylogroup B1, followed by A, D, and B2 [42,43,44]. Finally, to elucidate the evolutionary origin of camel uterine *E. coli* isolates. A phylogenomic tree was inferred for camel uterine *E. coli* isolates and pathotypes from extraintestinal and intestinal *E. coli* strains. The generated phylogenomic tree revealed two distant clades, representing intestinal and extraintestinal *E. coli* strains. The topology of the tree is in line with earlier *E. coli* pathotypes clustering [45]. The clustering of both CE isolates and non-CE isolates with intestinal *E. coli* suggests they evolved from intestinal *E. coli*. Notably, it was shown before that virulent extraintestinal isolates of *E. coli* often belong to phylogroups B2 and D, whereas intestinal isolates often belong to phylogroups B1, A, and C [46,47]. Another piece of evidence that our isolates evolved from intestinal *E. coli* is the fact that our camel isolates also belonged to phylogroups B1, A, and C.

HGT drives microbial adaptation and genetic diversity. Genes are mobilized by the interaction of numerous mobile genetic elements (MGEs) of different kinds [19,20]. We discovered a wide range of MGEs, including prophages, genomic islands, plasmids, and insertion sequences. Of the MGEs, genomic islands and plasmids carried several genes, including genes involved in metabolism, virulence, and antimicrobial resistance, which suggests that CE isolates and non-CE isolates had witnessed genetic transfer events.

Notably, 9–18% and 11–20% of the total genes in CE isolates and non-CE isolates, respectively, are attributable to HGT, suggesting that non-CE isolates have somewhat greater MGEs than CE isolates. This result is consistent with earlier studies that found 17.6% of the genes in *E. coli* were acquired through horizontal gene transfer [48]. Of note, the CE isolates 2649 and 2215A had a smaller genome size compared to other isolates, and this is due to missing several genes, including metabolic KEGG modules. CE isolates 2649 and 2215A may have lost several genes not needed in their previous habitat. Thus, isolates 2649 and 2215A show signs of genome reduction, which is similar to what has been observed in *Shigella* spp. [49].

The implications of these horizontally transferred genes resulted in wide variations in genome size and contributed to high genetic diversity observed among the isolates. Additional functional consequences of these horizontally transmitted genetic elements included the acquisition of virulence genes, increased capacity to utilize many carbon sources, and increased iron and nutrition absorption by cells via the ABC and PTS transporters. Finally, HGT played a role in the dissemination of antibiotic resistance determinants, leading to certain isolates being resistant to the trimethoprim, sulfonamide, streptomycin, and tetracycline antibiotics.

Given that five STs and seven serotypes were assigned to non-CE isolates and six STs and seven serotypes were assigned to CE isolates, we investigated if *E. coli* with the genotype or serotypes observed here had been detected in other infections before. We inquired for mentions of these STs in the literature, and we found the *E. coli* ST58, ST295, and ST173 that were identified during this study were mentioned in the literature as human uropathogens [50,51,52]. Although *E. coli* O103:H2 were identified in the literature as Shiga toxin-producing *E. coli* from human samples [53], they were shown to be Shiga toxin gene negative in the current investigation. Furthermore, *E. coli* O113:H21 was identified in the literature as an *E. coli* that produces Shiga toxin and is linked to hemolytic uremic syndrome and diarrhea in humans [54]. The Shiga toxin gene was present in *E. coli* O113:H21 in the current investigation. The Google Scholar search does not include information on the other genotypes and serotypes.

In order to adhere to host tissues and develop infection, *E. coli* needs adhesin proteins. All CE isolates and non-CE isolates had the adhesin curli fimbria gene (*csgA*), which is linked to the production of bacterial biofilms [55]. All CE isolates and non-CE isolates had the *nlpI* gene, which is necessary for intestinal epithelial cell invasion in Adherent Invasive *E. coli* strain LF82 [56]. Also, the fimbria operon *yehABCD* was present in 100% of CE isolates and non-CE isolates except one non-CE isolate lacking *yehD*. Along with adhesin proteins, hemolysin E (*hlyE*), a toxin that causes erythrocyte lysis and is cytotoxic to human macrophages, was present in all CE isolates and non-CE isolates [57]. Since the aforementioned adhesin and toxin genes are 100% prevalent and are known as virulent genes, it is likely that they reflect core genes rather than virulence factors. Given the aforementioned data, the virulence profile of *E. coli* by itself is insufficient to cause endometritis. Thus, host susceptibility and uterine microbiome composition should also be considered. Genomic studies revealed that certain genomic loci in the host influence uterine susceptibility to bacterial pathogens [58]. Data from the 16S rRNA metataxonomics research revealed that the uteri of pregnant cows and virgin heifers had microbiomes [59]. Additionally, the composition of the uterine microbiota in healthy and metritic cows is similar during the first two days following calving but then switches to increased richness of *Porphyromonas*, *Fusobacterium*, and *Bacteroides* in cows with metritis [60]. Fusobacterium was shown to be more abundant in the uterine microbiome of cows suffering from clinical endometritis and with the presence of *Trueperella* and *Peptoniphilus* [61]. Based on the aforementioned findings, endometritis is a polymicrobial illness caused by the coexistence of many uterine microorganisms that act synergistically to cause infection. Finally, a better understanding of endometritis will be possible when the data from host susceptibility and microbiome composition are combined.

In conclusion, the absence of *E. coli* genomic signatures from camels with or without clinical endometritis supports the non-specialization of *E. coli* strains in causing endometritis. Our findings also suggest that the camel uterine *E. coli* isolates probably evolved from intestinal *E. coli*.

Moreover, horizontal gene transfer driven by genomic islands and plasmids contributed to the genetic diversity of the isolates, resulting in the acquisition of virulence genes, metabolic characteristics, and antibiotic resistance determinants to trimethoprim, sulfonamide, streptomycin, and tetracycline.

## Figures and Tables

**Table 1:** General genomic features of *E. coli* isolates from camel uteri.

Strains	OrthoANI	Genome Size (bp)	Genome CDS	Plasmidome Size (bp)	Plasmidome CDS	Serogroup	ST	Phylogroup	Accession Number
CE isolates									
2649	99.6	4,369,294	4040	None	None	H33	13358	A	JBKIBG000000000
2215A	98.5	4,410,531	4069	16,186	20	O51:H15	5044	A	JBKIBF000000000
4280A	98.5	4,745,178	4418	None	None	O188:H7	180	B1	JBKIBJ000000000
1251A	98.5	4,853,119	4545	39,940	38	O8:H21	58	B1	JBKIBE000000000
1922big	98.5	4,986,463	4648	182,430	202	O167:H7	707	B1	JBKIBD000000000
4279	98.7	5,074,171	4764	129,640	142	O8:H4	88	C	JBKIBI000000000
3832	98.6	5,176,518	4898	144,257	155	O181:H49	173	B1	JBKIBH000000000
Non-CE isolates									
3275	98.5	4,632,655	4302	21,459	32	O50:H8	58	B1	JBKIBO000000000
2293	98.6	4,779,125	4481	74,255	83	O9:H12	58	B1	JBKIBK000000000
2613	98.6	4,790,025	4440	136,941	167	O188:H40	2520	B1	JBKIBN000000000
4436A	98.5	4,891,287	4577	6966	7	H11	295	B1	JBKIBQ000000000
3686A	98.5	4,947,877	4674	178,641	219	O74:H28	2164	B1	JBKIBP000000000
2387A	98.5	5,166,692	4925	64,099	71	O103:H2	17	B1	JBKIBL000000000
2571C	98.5	5,560,896	5213	69,119	86	O113:H21	3695	B1	JBKIBM000000000

**Table 2:** Antibiotic sensitivity results by the disk diffusion method and antibiotic resistance gene profile for *E. coli* isolates. Antibiotics highlighted in yellow mean the following (gentamicin, ceftazidime/avibactam, amikacin, ceftaroline, cefazolin ampicillin-sulbactam, amoxicillin-clavulanic, ceftiofur, meropenem, cefotaxime, and ceftriaxone). (+) indicates the presence of the gene, S indicates susceptible, and R indicates resistance.

Isolates	Typing			Disk Diffusion Method				Resistance Genes					
	Serogroup	ST	Phylogroup	Antibiotics	Ceftazidime	Streptomycin	Tetracycline	*strA*	*strB*	*dfrA1*	*dfrA5*	*sul2*	*tetA*
CE isolates													
2649	H33	13358	A	S	S	S	S						
2215A	O51:H15	5044	A	S	S	S	S						
4280A	O188:H7	180	B1	S	R	R	R	+	+	+		+	+
1251A	O8:H21	58	B1	S	S	S	S						
1922big	O167:H7	707	B1	S	S	S	R						+
4279	O8:H4	88	C	S	S	R	R	+	+			+	+
3832	O181:H49	173	B1	S	S	S	R				+		+
Non-CE isolates													
3275	O50:H8	58	B1	S	S	S	S						
2293	O9:H12	58	B1	S	R	S	S						
2613	O188:H40	2520	B1	S	R	R	R	+	+	+		+	+
4436A	H11	295	B1	S	S	S	S						
3686A	O74:H28	2164	B1	S	S	S	S						
2387A	O103:H2	17	B1	S	S	S	S						
2571C	O113:H21	3695	B1	S	S	S	S						

**Table 3:** Virulence genes predicted in uterine *E. coli* isolates from camels. The existence of the virulence trait is shown by the yellow box. A lack of the virulence trait is shown by a white box.

Strain		4436A	2571C	2613	3686	2293	3275	2387A	3832	4280A	1922big	1251A	2215a	4279	2649
Group		Non-CE isolates							CE isolates						
MST typing		295	3695	2520	2164	58	58	17	173	180	707	58	5044	88	13358
Phylogroup		B1	B1	B1	B1	B1	B1	B1	B1	B1	B1	B1	A	C	A
Adhesin	*csgA*														
	*fdeC*														
	*fimH*														
	*hra*														
	*iha*														
	*lpfA*														
	*nlpI*														
	*tir*														
	*yehA*														
	*yehB*														
	*yehC*														
	*yehD*														
Invasion	*AslA*														
	*tia*														
Iron uptake	*fyuA*														
	*ireA*														
	*iroN*														
	*irp2*														
	*iucC*														
	*iutA*														
	*sitA*														
Toxins	*astA*														
	*eae-e01-epsilon*														
	*hlyE*														
	*hlyF*														
	*senB*														
	*stx2b-O174-031*														
Protectins	*capU*														
	*cba*														
	*cea*														
	*cia*														
	*cma*														
	*colE7*														
	*cvaC*														
	*espA*														
	*espB*														
	*espF*														
	*espJ*														
	*etpD*														
	*etsC*														
	*hha*														
	*iss*														
	*mchB*														
	*mchC*														
	*mchF*														
	*mcmA*														
	*ompT*														
	*shiA*														
	*shiB*														
	*traJ*														
	*traT*														
Others	aamR:FN554766														
	*anr*														
	*gad*														
	*nleA*														
	*nleB*														
	*tccP*														
	*terC*														
% genes in GIs		40	42.9	53.8	37.5	50	30.8	65.4	78.8	46.2	63.2	58.8	57.1	64.3	33.3

## Data Availability

The sequenced genomes of *E. coli* in the current study were deposited in GenBank.

## Supplementary Materials

The following supporting information can be downloaded at: https://www.mdpi.com/article/10.3390/vetsci12050457/s1, Table S1: Pairwise ANI values of *E. coli* isolates; Table S2: General characteristics of toxin-antitoxin systems in uterine *E. coli* strains; Table S3: Mobile elements. Blue box indicates presence of mobile element; Table S4: The number and size of pathogenicity islands, as well as the virulence factors within pathogenicity islands.
