# Supplementary material for: No Genomic Signatures Were Found in Escherichia coli Isolates from Camels With or Without Clinical Endometritis
Source: Vet Sci. 2025 May 10;12(5):457. doi: 10.3390/vetsci12050457 (PMC12115863; doi:10.3390/vetsci12050457)
Supplement: Supplementary file 1 [file vetsci-12-00457-s001.zip › vetsci-3624903-supplementary.pdf]

Supplementary Table S1. Pairwise ANI values of *E. coli* isolates. Numbers are displayed as percentages in the matrix cells. The yellow colour represents ANI value above 99% while blue colour represents ANI value less than 99%.

**Non-CE isolates**	**3275**	**2293**	**2613**	**4436A**	**3686A**	**2387A**	**2571C**
**3275**	100.0	99.7	99.2	99.2	99.1	99.1	99.3
**2293**	99.7	100.0	99.0	99.0	99.0	99.0	99.1
**2613**	99.2	99.0	100.0	99.1	99.1	99.0	99.1
**4436A**	99.2	99.0	99.1	100.0	99.1	99.0	99.0
**3686A**	99.1	99.0	99.1	99.1	100.0	98.8	99.0
**2387A**	99.1	99.0	99.0	99.0	98.8	100.0	98.8
**2571C**	99.3	99.1	99.1	99.0	99.0	98.8	100.0
**CE isolates**	**2649**	**2215A**	**4280A**	**1251A**	**1922big**	**4279**	**3832**
**2649**	100	98.4	98.5	98.5	98.5	98.6	98.6
**2215A**	98.4	100	98.3	98.3	98.3	98.4	98.4
**4280A**	98.5	98.3	100	99.1	99.3	98.8	99.1
**1251A**	98.5	98.3	99.1	100	99.1	98.8	99.1
**1922big**	98.5	98.3	99.3	99.1	100	98.9	99.0
**4279**	98.6	98.4	98.8	98.8	98.9	100	98.9
**3832**	98.6	98.4	99.1	99.1	99.1	98.9	100

Supplementary Table S2. General characteristics of toxin-antitoxin systems in uterine E. coli strains. Yellow colour indicates the presence of the toxin-antitoxin gene sets while red colour indicates absence of the toxin-antitoxin gene sets. HGT indicate genes acquired horizontally.

Type TA system	TA system	4436A	2571C	2613	3686	2293	3275	2387A	3832	4280A	1922big	1251A	2215a	4279	2649
Type I	Hok-Sok	HGT	HGT	HGT	HGT			HGT			HGT	HGT		HGT	
Type II	Hha-TomB							HGT	HGT					HGT	
	HigB-HigA				HGT	HGT	HGT		HGT			HGT	HGT		
	MazF-MazE														
	PspC-PspB														
	VapC-VapB	HGT		HGT	HGT	HGT						HGT	HGT		
	HipA-HipB				HGT		HGT			HGT					HGT
	YafQ-dinJ														HGT
	YhaV-PrlF				HGT										
	YoeB-YefM		HGT												
	ParE-ParD	HGT	HGT	HGT					HGT		HGT			HGT	
	RelE/StbE-RelB/StbD	HGT		HGT		HGT	HGT	HGT		HGT	HGT	HGT		HGT	
	HicA-HicB				HGT										
	ChpB-ChpS														
	CcdB-CcdA			HGT	HGT	HGT					HGT	HGT			
	MqsR-MqsA														
	YafO-YafN		HGT												HGT
	Doc-Phd			HGT	HGT						HGT				
Type IV	YeeV-YeeU	HGT	HGT	HGT		HGT	HGT	HGT	HGT	HGT	HGT	HGT	HGT	HGT	HGT
	CptA-CptB														
Type V	GhoT-GhoS				HGT									HGT	
	**Total TA system**	17	15	18	17	15	16	16	13	15	17	16	16	15	16
	**Total HGT TA system**														

Supplementary Table S3. Mobile elements. Blue box indicates presence of mobile element.

Group		CE isolates							Non-CE isolates						
Strains		1922bi	1251A	2215A	2649	4280A	4279	3832	3275	2293	2613	4436A	3686A	2387A	2571C
Insertion Sequences	cn_10071_IS682											■			
	cn_1489_ISEc1					■									
	cn_17025_IS911							■							
	cn_30268_IS609														■
	cn_43524_IS609														■
	cn_5813_IS911						■								
	cn_8136_ISEc1						■								
	IS100	■	■	■	■		■		■	■				■	
	IS102					■				■					
	IS26			■			■	■							
	IS3						■						■	■	
	IS30									■	■			■	■
	IS4			■			■								
	IS421			■	■										
	IS609	■	■	■	■	■	■	■	■	■	■	■	■	■	■
	IS629		■			■	■	■		■		■	■		
	IS640									■					
	IS679	■	■									■	■		
	IS682		■							■		■			
	IS903			■											
	IS911					■	■	■							
	ISEc1		■		■	■	■	■	■			■	■	■	■
	ISEc11											■			
	ISEc13							■							■
	ISEc30			■											
	ISEc31							■	■	■		■		■	■
	ISEc32							■							
	ISEc38				■	■	■	■	■	■				■	
	ISEc45														■
	ISEc52			■											
	ISEc78					■									■
	ISEsa1		■			■			■	■		■			
	ISKpn26	■													
	ISKpn8			■	■										
	ISSen1												■		

	ISSfl10														
	ISSfl3														
	ISSfl8														
	MITEEc1														
Plasmid Replicon Type	Col(MG828)														
	Col156														
	ColpVC														
	IncFIA														
	IncFIB(AP001918)														
	IncFII														
	IncFII(29)														
	IncFII(pCoo)														
	IncFII(pHN7A8)														
	IncI1														
	IncQ1														
	IncR														
	IncX1														
	IncY														
GI Prophage	Total CDS	687	642	370	407	490	750	884	511	674	604	689	720	829	1064
	Number	2	3	2	1	3	6	7	2	3	4	3	5	8	8

Supplementary Table S4. The number and size of pathogenicity islands, as well as the virulence factors within pathogenicity islands.

Strain	Pathogenicity islands		
	Name	Size (kb)	Virulence factors and resistance genes within Pathogenicity islands
2649	PAI2649	6.4	*yehA, yehB, yehC, yehD*
4279	PAI4279-1<br>PAI4279-2<br>PAI4279-3	5.8<br>174<br>49	*yehA, yehB, yehC, yehD*<br>*ompT, iss, hlyF,etsC, iroN, mchF, cvaC, cia, anr, traT ,traJ, iutA, iucC, strA, strB, sul2 and tetA*<br>*iss*
2215A	PAI2215A-1<br>PAI2215A-2<br>PAI2215A-3	5.8<br>72.4<br>31	*yehA, yehB, yehC, yehD*<br>*iss, ompT*<br>*irp2, fyuA*
1251A	PAI1251A-1<br>PAI1251A-2<br>PAI1251A-3<br>PAI1251A-4<br>PAI1251A-5	5.8<br>22<br>4<br>21<br>113	*yehA, yehB, yehC yehD*<br>*ompT*<br>*hlyE*<br>*astA*<br>*traJ, traT, anr*
1922big	PAI1922big-1<br>PAI1922big-2<br>PAI1922big-3	5.8<br>7.9<br>18<br>179<br>85	*yehA, yehB, yehC, yehD*<br>*ompT*<br>*iss*<br>*cba, cma, traT, tetA*<br>*cea, colE7, senB,*

	PAI1922big-4<br>PAI1922big-5		
4280A	PAI4280A-1<br>PAI4280A-2<br>PAI4280A-3<br>PAI4280A-4	5.8<br>42.5<br>193<br>15.4	*yehA, yehB, yehC, yehD*<br>*iss*<br>*capU*<br>*strA, strB, dfrA1, sul2 and tetA*
3832	PAI3832-1<br>PAI3832-2<br>PAI3832-3<br>PAI3832-4<br>PAI3832-5	5.8<br>59.7<br>6.8<br>214<br>89	*yehA, yehB, yehC, yehD*<br>*ompT, iss*<br>*iha*<br>*iha, cia, cvaC, mchF, iroN, etsC, hlyF, sitA, ompT, iss, iucC, iutA, traJ, traT, cea, mchB, mchC, mcmA, anr*<br>*tetA, dfrA5*
2571C	PAI-2571C-1<br>PAI-2571C-2<br>PAI-2571C-3<br>PAI-2571C-4<br>PAI-2571C-5<br>PAI-2571C-6	5.8<br>6<br>131.4<br>120<br>33.8<br>126	*yehA, yehB, yehC, yehD*<br>*ompT*<br>*ireA*<br>*stx2, stx2b-O174-031, hha*<br>*cea*<br>*mR:FN554766*
2613	PAI-2613-1<br>PAI-2613-2<br>PAI-2613-3<br>PAI-2613-4	5.8<br>5<br>75<br>15.4	*yehA, yehB, yehC, yehD*<br>*lpfA*<br>*traJ, traT*<br>*strA, strB, dfrA1, sul2 and tetA*
3686A	PAI-3686A-1<br>PAI-3686A-2	5.8<br>107	*yehA, yehB, yehC, yehD*<br>*anr, astA*
4436A	PAI-4436A-1<br>PAI-4436A-2	5.8<br>126	*yehA, yehB, yehC, yehD*<br>*traT, anr*
2293	PAI-2293-1<br>PAI-2293-2<br>PAI-2293-3	5.8<br>64.8<br>118	*yehA, yehB, yehC*<br>*iss*<br>*traJ, traT, anr*
3275	PAI-3275	5.8	*yehA, yehB, yehC, yehD*
2387A	PAI-2387A-1<br>PAI-2387A-2<br>PAI-2387A-3<br>PAI-2387A-4<br>PAI-2387A-5	5.8<br>10<br>42<br>188<br>5<br>40	*yehA, yehB, yehC, yehD*<br>*nleB*<br>*iss*<br>*anr, traT, ompT, hha, etpD*<br>*tccp*<br>*espF, espB, espA, eae-e01-epsilon, tir*

	PAI-2387A-6		
